# Social Networks Play a Complex Role in HIV Prevention Knowledge, Attitudes, Practices, and the Uptake of PrEP Through Transgender Women Communities Centered Around Three “Casas Trans” in Lima, Peru: A Qualitative Study

**DOI:** 10.1177/23259582231196705

**Published:** 2023-09-27

**Authors:** Tijana Temelkovska, Kathleen Moriarty, Leyla Huerta, Amaya Perez-Brumer, Eddy Segura, Ryan Colby Passaro, Jordan E Lake, Jesse Clark, Cherie Blair

**Affiliations:** 1 David Geffen School of Medicine at UCLA, Los Angeles, CA, USA; 2 Department of Obstetrics and Gynecology, University of Colorado Anschutz, Aurora, CO, USA; 3 71541 Department of Internal Medicine, Medstar Georgetown University Hospital, Washington, DC, USA; 4 Feminas, Lima, Peru; 5 Division of Social and Behavioral Health Sciences, Dalla Lana School of Public Health, University of Toronto, Toronto, Canada; 6 Facultad de Ciencias de la Salud, Universidad de Huánuco, Huánuco, Perú; 7 Department of Emergency Medicine, Keck School of Medicine at the University of Southern California, Los Angeles, CA, USA; 8 Department of Internal Medicine, McGovern Medical School at UTHealth, Houston, TX, USA; 9 Department of Medicine, Division of Infectious Diseases, David Geffen School of Medicine at UCLA, Los Angeles, CA, USA

**Keywords:** transgender women, Peru, HIV prevention, PrEP, social network

## Abstract

Transgender women's (TW) social networks may facilitate HIV prevention information dissemination and normative reinforcement. We conducted a qualitative study of social networks among 20 TW affiliated with 3 “casas trans” (houses shared among TW) in Lima, Peru, using diffusion of innovations theory to investigate community-level HIV prevention norms. Participants completed demographic questionnaires, social network interviews, and semistructured in-depth interviews. Median age was 26 and all participants engaged in sex work. Interviews revealed high HIV prevention knowledge and positive attitudes, but low engagement in HIV prevention. Respondents primarily discussed HIV prevention with other TW. Network members’ opinions about pre-exposure prophylaxis (PrEP) frequently influenced respondents’ personal beliefs, including mistrust of healthcare personnel, concern that PrEP efficacy was unproven, fear of adverse effects, and frustration regarding difficulty accessing PrEP. Patterns of influence in TW networks may be leveraged to improve uptake of HIV prevention tools, including PrEP.

## Introduction

Globally, transgender women (TW) face a disproportionate HIV burden.^1,2^ In Peru and other Latin American countries, the HIV epidemic is concentrated among men who have sex with men (MSM) and TW, with prevalence rates between 30% and 48% among TW.^1,3–7^ It has been extensively documented that TW experience social exclusion, economic marginalization, and stigma at the societal, institutional, and individual levels, which create barriers to accessing healthcare, including HIV services.^8–11^

HIV pre-exposure prophylaxis (PrEP) is an effective prevention tool with the ability to put the power of HIV prevention in the hands of TW.^12^ However, there is currently a lack of widespread PrEP implementation among TW due to persistent challenges related to PrEP access, acceptability, and adherence among TW.^13^ Several studies have found PrEP acceptability to be low among TW, primarily due to mistrust of medical establishments and prioritization of feminizing hormone use.^14^ These factors likely contribute to significantly lower PrEP adherence among TW, as demonstrated by the subgroup analysis of TW enrolled in the iPrex trial.^15^ Within the Peruvian healthcare system specifically, barriers to PrEP access include lack of trans-specific, trans-sensitive care, lack of trans-inclusive PrEP marketing, and lack of organized PrEP access or programming.^14,16–18^ Peru does not currently have a public PrEP program; instead, PrEP is primarily available through demonstration projects and research studies. Though it can be accessed through certain clinics, the cost is prohibitively expensive for many TW who may already experience economic insecurity and marginalization.^16^

Social networks provide a potential avenue for raising awareness and improving acceptability of PrEP among hard-to-reach populations. Several studies among MSM in the United States have demonstrated that social networks are key sources of PrEP information and support for MSM.^19,20^ Peer navigation has also shown promise for linking individuals to HIV prevention services and PrEP, especially for MSM and TW experiencing multiple syndemic health disparities.^21–23^

More research is needed to understand the role of TW social networks in shaping HIV prevention knowledge, attitudes and practices and possible implications for PrEP programming. Key components of HIV risk include individual behavior, social and sexual network interactions and influences, and structural contexts. TW social networks play an important role in structuring HIV prevention norms across all these components. Several studies have shown that TW's social networks influence individual behavior, including involvement in sex work, feminizing hormone use, and HIV prevention practices.^24–27^ TW social networks have also been shown to be important for disseminating information. A study of TW sex workers revealed that respondents preferred to receive information from other TW sex workers or community members as opposed to medical institutions.^28^ Support and social cohesion from TW networks may also represent an important source of social capital that can help TW overcome barriers and stigma to accessing healthcare, create alternative systems to access care, and strengthen advocacy within TW communities.^8^ As such, TW networks may play an important role in PrEP uptake and diffusion through TW communities.

Diffusion of innovations (DOI) theory underscores the role of social networks in the adoption of an innovation.^29^ Key concepts of this framework that can be applied to the diffusion of HIV prevention practices in social networks include the concepts of homophily, opinion leadership, and perceived innovation attributes. The DOI model has been used to guide HIV prevention efforts in diverse settings and several concepts of this model have emerged as particularly valuable for the adoption of HIV preventive behaviors, especially opinion leadership.^30,31^ However, previous unsuccessful attempts to utilize DOI theory, specifically through leveraging opinion leaders, in HIV prevention strategies in Peru may call into question the utility of this approach. The National Institute of Mental Health (NIMH) Collaborative HIV/STD (sexually transmitted disease) prevention trial was a 10-year, multicountry group-randomized trial of a community popular opinion leader (C-POL) intervention guided by DOI concepts.^32^ This intervention failed to show any difference in sexual risk practices and sexually transmitted infection (STI) rates between the C-POL intervention group and the AIDS education comparison group.^32^ In Peru specifically, rates of reported unprotected sexual intercourse (behavioral outcome) and of STI acquisition (biological outcome) were both slightly lower in the comparison group compared to the C-POL intervention group, with no statistically significant difference found.^32^ Though these results could be interpreted as a failing of DOI theory in this context, it is important to note that in this study, the C-POL intervention group was tested against a comparison group that still received a robust education program on the prevention of HIV and STIs that garnered media attention and likely contributed to increased overall public health awareness within these communities.^32^ It is likely that as a result, both C-POL and comparison groups experienced increases in HIV/STI prevention messaging that permeated through social networks and impacted social norms. In Peru, though there was no difference between intervention and comparison group in this trial, both groups saw an improvement in behavioral and biological outcomes compared to baseline.^32^ Furthermore, this C-POL intervention was designed based on successful experiences in small US cities and cultural differences may have made the intervention difficult to generalize to other contexts, such as the selected *barrios* in Peru in this trial. The use of C-POLs and other DOI concepts may still have promise if these frameworks are carefully adapted to local contexts. Finally, the C-POL intervention was specifically designed to increase discussions about HIV/STI prevention, and thus knowledge, while the measured outcome was a change in actions and practices (ie, sexual risk reduction). This highlights a recognized pitfall of DOI-based intervention: the assumption that providing information alone will lead to changes in behavior or practice.^33^ This may instead lead to a gap between knowledge and attitudes regarding an innovation, and actual practice (knowledge, attitudes, and practice-gap or KAP-gap).

The KAP-gap has been described at length, particularly in attempts to understand the discrepancy between family planning knowledge or values related to future pregnancy and actual use of contraceptive methods, but can be applied to many public health interventions.^34,35^ Importantly, the KAP-gap highlights that increasing knowledge and changing attitudes alone may not correlate with behavioral change. However, DOI theory can be used to target interventions specifically toward the goal of changing practice and closing the KAP-gap by utilizing opinion leaders to model behavioral change, for example. Several interventions have demonstrated the utility of this approach across a variety of global contexts. In Haiti, a DOI-based intervention successfully leveraged the authority of village voodoo practitioners to actively encourage attendance of USAID-sponsored HIV/AIDS education meetings.^36^ In Nepal, an intervention aimed at addressing maternal and child vitamin A deficiency used social modeling to encourage planting of kitchen gardens of fruits and vegetables.^36^

We used DOI theory as a framework to understand potential structures for the uptake and dissemination of PrEP as an HIV prevention strategy within TW social networks in Lima, Peru. Examining the current challenges to PrEP uptake through the lens of key DOI concepts, such as homophily and opinion leadership, may contribute to an improved understanding of potential approaches to improve PrEP diffusion. In addition, DOI theory highlights 5 perceived attributes of an innovation that impact the pace of its diffusion including an innovation's (1) relative advantage, or the perceived benefits of the innovation; (2) its trialability, or the degree to which it can be tested on a limited basis prior to adoption; (3) its observability, or the extent that the innovation's effects can be seen by adopters; (4) its complexity, or the perceived difficulty of using or adopting the innovation; and (5) its compatibility, or the extent to which an innovation fits the values and needs of the adopters.^29,37^ Understanding how barriers to PrEP uptake fit within this framework can support future PrEP interventions and research that utilizes this theory could help inform PrEP interventions that are adapted to the local contexts of TW communities and leverage existing network structures to facilitate diffusion of PrEP.

Houses shared among TW represent a key structural organizing framework for the lives of TW through which the diffusion of an innovation like PrEP might flow. Data from residents of 3 *casas trans* in Lima, Peru, was used to explore how TW social networks contribute to HIV prevention knowledge, attitudes and practices, especially regarding PrEP. We sought to evaluate the potential role of the networks of TW living in or associated with these *casas trans* in the diffusion of PrEP throughout this community of TW.

## Methods

### Participants and Recruitment

Data was collected in May 2018 in the greater Lima area. Participants were selected using convenience sampling. Participants were recruited from a local TW community organization in Lima. Participants were eligible if they were 18 years or older, self-reported HIV negative or serostatus unknown, self-identified as a transgender woman (*transgénero, travesti, mujer trans)*, and had engaged in anal or oral sex in the past 12 months. Twenty-two interested TW were screened and 20 were eligible for the study. One TW was under 18 years old, and 1 TW had not engaged in anal or oral sex in the past 12 months.

### Study Procedures

All participants provided written informed consent and completed a brief demographic questionnaire, a standardized social network interview, and semistructured qualitative interview. Two interviewers fluent in Spanish conducted the interviews; one interviewer was a member of the TW community in Lima and the other interviewer was a cis-woman researcher experienced in conducting qualitative interviews on issues related to HIV and STIs. Interviews were conducted in a private and discreet location. The interview guide was created with input from 2 members of a TW community organization in Lima. The guide was then piloted with 3 TW and modified based on results of this pilot. During qualitative interviews, respondents were asked to list the 3 closest or most influential individuals in their network and probes assessed relationship dynamics, types of support received, communication patterns, knowledge and attitudes regarding condom use and PrEP, HIV/STI discussions, and how these discussions influence respondents’ attitudes and behaviors. After each qualitative interview, the interview was reviewed to identify preliminary thematic codes, and recruitment ended once thematic saturation of qualitative interviews was reached. Interviews were audio transcribed and checked for completeness and accuracy by a member of the research team. Each interview lasted between 45 and 60 min. Participants were compensated 40 *Nuevos soles* (approximately 13 USD) for their time.

### Data Analysis

Quantitative demographic and social network data was analyzed using Stata. Qualitative data from the semistructured interview was analyzed in Atlas.ti using immersion-crystallization, a process that allows the researchers to immerse themselves in the data and reflect on themes that arise from the immersion process.^38^ A preliminary codebook was developed using a deductive approach based on the interview guide and anticipated themes from literature review. Two researchers initially read through the transcripts once to identify major themes in order to determine when thematic saturation was achieved. The preliminary codebook was then used by one researcher to code all transcripts line by line. Codes were further refined during this process using an inductive approach that included addition of codes for themes not yet represented and merging certain codes as relevant to the theoretical frameworks guiding the analysis. Qualitative analysis was guided by DOI and KAP-gap theories.^29^

### Ethical Approval and Informed Consent

The study was approved by the Ethics Committee at Asociación Civil Impacta (0014-2018-CE) and the Institutional Review Board at the University of California, Los Angeles (17-001538-CR-00001) prior to beginning any study procedures. All study procedures adhered to the standards set forth by the Declaration of Helsinki. Informed consent was obtained from all participants included in the study prior to the initiation of study procedures.

## Results

### Demographic Information

Twenty TW residing in or affiliated with 1 of 3 “*casas trans*” (houses shared among TW) in Lima, Peru, participated in this study. Respondents had a median age of 26 years. Three-quarters of respondents were born outside of Lima and had lived in Lima for a median of 5 years. Seventeen respondents (85%) were residing in a “*casa trans*” at the time of data collection. All respondents engaged in sex work. Thirteen respondents (65%) were in a relationship. Eleven (55%) reported “always” using condoms. Seventeen (85%) had heard of PrEP, but only 3 (15%) used PrEP (Table 1).

**Table 1.** Participant Characteristics and Demographic Information.

Characteristics	Participants (N = 20)
	n (%)
Age, median (IQR)	26 (21.5-32.5)
Region of birth	
Lima/Callao	5 (25)
Northern coastal	5 (25)
Amazon	9 (55)
Andes	9 (5)
Years in Lima^a^, median (IQR)	5 (1.6-7)
Currently living in trans house	17 (85)
Education	
Did not complete secondary school	9 (45)
Completed secondary school or greater	11 (55)
Household income^b^	
300-500 Soles/month	2 (10.5)
501-1500 Soles/month	16 (84.2)
1501-3000 Soles/month	1 (5.3)
Relationship status	
Single	13 (65)
In a partnership	7 (35)
Sexual role^3^	
Passiva	12 (63.2)
Moderna	7 (36.8)
Condom use	
Always uses condoms	11 (55)
Does not always use condoms	9 (45)
PrEP awareness	
Aware of PrEP	17 (85)
Uses PrEP	3 (15)

IQR: interquartile range; PrEP: pre-exposure prophylaxis.

^a^ If born in other region (n = 15).

^b^ N = 19 due to missing data.

### TW Play an Important Role in HIV-Prevention Knowledge Sharing Among Their Networks

Respondents had a high degree of awareness regarding the importance of condom use and routine HIV/STI testing and reported that this information was circulated within their networks. TW played a key role in sharing knowledge and disseminating information regarding condom use and routine HIV/STI testing. Within TW networks, older or more experienced TW often educated younger or newly arrived TW, such as those that were new to the city and/or the profession of sex work, on the importance of these prevention measures. Several respondents reported that the friend who introduced them to sex work and this community of TW also taught them about prevention.

“She [TW friend] told me, ‘you know what, let's go [to Lima] to work’, she explained to me and we bought condoms and all that, she told me that I needed to use [condoms], and also [other TW friend] since she has been there a while, she has advised me.”—*22 years old*

“When I started working in this [sex work]… a trans friend [told me] that I always have to use condoms, always in this routine that I have, I have to use them… because it is sex work.”—*31 years old*

TW also shared knowledge throughout other networks, such as with family or partners, where respondents primarily acted as the source of HIV prevention information. For example, almost all partnered respondents discussed having more HIV prevention knowledge than their partners and needing to teach partners about the importance of condom use and routine testing.

Interviewer Do you trust his [partner's] opinions about health?

Respondent Well, him not so much because I have more [knowledge] from [sex work] so I know more than him.

Interviewer You know more than him about health?

Respondent Of course. About how you can contract a venereal disease and all of that… because when you go through your controls (routine HIV/STI testing), they teach you a lot about this, about all the different types.—*35 years old*

“He [partner] did know about HIV but didn’t know some things that, I from experience, know a ton. About precautions…about how to avoid contracting [HIV].”—*38 years old*

A few respondents even shared this information with younger family members, such as siblings or nieces and nephews, to teach them about HIV/STI prevention.

“I also told [my sister], ‘you have to use a condom so that you don’t get pregnant, another reason is that there are plenty of STIs, sexually transmitted infections like HIV, the condom isn’t just for pregnancy, but also for other risks that you need to protect yourself from,’ I told her.”*—23 years old*

Some also discussed these issues with supportive family members to educate them about HIV prevention and reassure family members that they are taking measures to mitigate risks.

“I have explained to her [mother] the risks that I’m exposed to from working in the street… I tell her that there are various sexually transmitted diseases like HIV, AIDS, syphilis, so many diseases that there are.”—*27 years old*

Within networks of TW, relatively less knowledge was circulated about PrEP. While every respondent reported discussing condom use with their TW friends, discussions surrounding PrEP were much less common and often defined by misinformation. Consequently, accurate PrEP knowledge was much lower among these networks as demonstrated by several respondents’ reports that they did not have a clear understanding of how PrEP functioned or what effects to expect from taking PrEP.

“I think that in some people, it can be that they are at a disadvantage if they take [PrEP], and it's like it freezes their virus, or I don’t know… It would make me afraid, because, I mean, let's say that I come to be in the window period because of [partner living with HIV], you understand? And I take PrEP, and my virus I will have like this, I mean like encapsulated, you understand?”—*26 years old*

“I was afraid, because the pill they gave me for syphilis bothered me and…I don’t know how it can evolve in my body… I don’t know how it will react in my body because pills bother me.”—*26 years old*

### Relationships Between Trans Women's HIV Prevention Attitudes and Practices Varied by Different HIV Prevention Modalities, Were Influenced by Their Social Networks, and Were Often Mediated by Barriers Outside of Their Control

#### Routine HIV/STI Screening was Viewed Positively by TW Networks and Commonly Accessed With Network Support

Respondents’ discussions with others in their network, including family, partners, and other TW, reflected universally positive attitudes toward routine HIV/STI testing. In addition to sharing positive attitudes, respondents reported regularly accessing routine HIV/STI screening. TW networks were important for facilitating HIV testing access, as some respondents described getting tested together with their TW friends.

“In the work that I do, every 6 months I do my analysis… one day a trans came… she came and she told me, let's go [get tested] and I went.”—*19 years old*

“Always with [TW friend], we always do our controls [HIV/STI screening] together.”—*26 years old*

Others who routinely accessed HIV/STI testing reported bringing their primary partners to get tested as well. As such, respondents were often the source of HIV/STI screening knowledge and access in other relationships outside the TW community.

“I also tell him [partner] that he should go get the tests for HIV, syphilis, I always tell him this and he says ‘yes’ and he comes with me sometimes to do it.”—*22 years old*

#### While Respondents’ Networks of Family and TW Friends Held Positive Attitudes Toward Condoms as HIV Prevention Tools, TW's Sexual Partners Were Often the Network Members Responsible for Dictating Actual Condom Use

The public discourse surrounding condom use—including both what was stated during interviews and what was reported to be shared informally among TW—reflected positive attitudes and encouragement.

“She [TW friend] always tells me to take care of myself, she's always ‘don’t be with anyone without a condom’, or rather, we both give each other advice.”—*21 years old*

These positive attitudes were also reflected in the few conversations that respondents had about these topics with family. However, there was an observed difference between discussions and actions, whereby the discourse and attitudes shared among TW and with family were not reflected in the actions experienced with sexual partners. Despite the support for condom use among respondents’ close network members, in practice, condoms were not used regularly by respondents. Instead, the power and influence exerted by respondents’ cis-male sexual partners often governed actual condom use practices. For example, respondents with primary partners reported not using condoms consistently in these relationships.

“When we had just started [going out], for almost one year we used [condoms], when we met in Pucallpa, for one year we used them.. and since then, not anymore.”—*36 years old*

Interviewer And with [primary] partners do you also use condoms?

Respondent No… I don’t know, I think it's because there is trust.—*27 years old*

Though many partnered respondents reported engaging in condom discussions with their primary partners, they typically accepted that condoms would not be used in their partnership and instead focused negotiations on emphasizing condom use with any concurrent partners. Respondents, who were more knowledgeable about HIV/STIs, carried the responsibility of educating partners about prevention and sometimes found it difficult to stress the importance of condom use to their partners.

“I’ve told him clearly, ‘if you are cheating on me, use condoms. Because if you’re going to cheat on me, you’re going to harm me, you mess with who you mess with… and if you do it without a condom, you have to tell me so as to not put me at risk or infect me with something.”—*33 years old*

Furthermore, some respondents may have felt that they did not have the power to engage in these negotiations with partners.

Interviewer Have you talked about using condoms with your partner?

Respondent What happens is that I don’t use it with him, really.

Interviewer And why?

Respondent I don’t know, it bothers him or he doesn’t like it… he doesn’t want to.—*35 years old*

“I always want to protect myself, but he does not really agree… he gets annoyed or is super jealous, or things like this.”—*38 years old*

Sexual partnerships with sex clients also represented barriers to condom use for respondents. These relationships were characterized by power imbalances and economic coercion. Respondents reported that clients often offered more money for condomless intercourse and though respondents publicly denounced this practice, many reported being influenced by the monetary incentive or knowing other TW who accepted these offers.

“She told me, I don’t know if this was true or a joke, but she says… ‘if he [client] doesn’t want to [use a condom], I’m not going to lose my 30 soles, my 40 soles,’ she tells me…”—*19 years old*

“It was two times, nothing more [having sex without a condom]. But when I do oral, it's like you suck on the condom and sometimes I don’t want to and I get carried away by the money; they [clients] don’t like to put on the condom, it's like they like it like this.”—*24 years old*

“This has happened to me, I have not used [a condom] because they paid me a little more… but these are very few times… Sometimes between us we know ‘Ay! These clients want to fuck without a condom or they want to penetrate you without a condom.’”—*27 years old*

Other times, even when respondents made efforts to use condoms, sex clients were reported to engage in sexually violent behaviors such as removing condoms during intercourse without consent.

“There have been other times where clients come, they offer more, or sometimes they don’t even offer and they want it without a condom. It's happened to me also that they take it off… I realize and I get upset and I don’t let it. I mean, I don’t let it, no, I’m going to put it on, forget that.”—*20 years old*

“With my clients I never stop using [condoms]… I use them all the time… Including sometimes during the penetration there are clients that sometimes will remove the condoms.”—*36 years old*

Overall, despite widespread TW network support for condom use, a gap between condom use knowledge, attitudes, and practices persisted and was mediated by barriers to condom use which were outside of respondents’ control. Several respondents recognized this disparity between what is publicly discussed among TW and what is actually put into practice.

“She [TW friend] told me that she was with a client and the client wanted something more, or rather, without a condom. I advise her, but she makes the decision, because I don’t see her between 4 walls what she does with the client.”*—35 years old*

#### Respondents’ Social Networks Played a Significant Role in Influencing Both PrEP Attitudes and Practices

The majority of respondents that discussed PrEP with others in their network described attitudes that reflected hesitancy and mistrust. Both PrEP attitudes and practices were heavily influenced by the relative limitation of PrEP access to research study participants. This obstacle tended to perpetuate mistrust of the medical field, research personnel, and the safety and effectiveness of PrEP for TW communities. As a result, common attitudes that circulated regarding PrEP included notions that PrEP had not been scientifically proven yet or that research personnel were experimenting on TW. These attitudes prevailed among diverse network members including other TW, partners, and family.

“She [mother] told me that it's great that there is a cure for HIV, no? but still, that I don’t take it because it's something that is not scientifically proven 100%. ‘No *marabunda*, no, you no’. And I also opted for no.”*—38 years old*

Respondent That it's good for us? No. In the first place, we’re young. I recently turned 19 years and there are people who have passed away at 17, 16 years…I’m going to doubt [PrEP] until the day that… until it truly is for consumption and should prevent the diseases.

Interviewer Have you ever talked to a doctor to ask about the pill?

Respondent No.—*19 years old*

“I don’t believe in this pill, for me it is an experiment, it is still not proven that this is a pill that is correct for us to take and to not take care of ourselves anymore… I heard a conversation of a girl. I passed by and told her then that the pill that is being given now is a lie because she says that she took it, the girl, and she didn’t take care of herself [use a condom], and she went to take a test at 6 months, and she had a positive [HIV] result.”—*19 years old*

Respondents’ attitudes regarding PrEP were often influenced by attitudes circulated within their networks. For example, some respondents described changing their opinion about PrEP after another network member voiced PrEP hesitancy and mistrust of PrEP studies.

Respondent The only thing he [partner] told me, he just told me ‘be careful that they are doing an experiment in your body, I don’t want you to have problems later’… I told him, well, that this helps us to take care of ourselves much more and he says ‘that's what you say, but imagine that they are deceiving you and giving you things that shouldn’t be.’

Interviewer His opinion changed your opinion a little or no?

Respondent Yes, the truth is yes, also as they told us about the infusions, that also, he told me ‘you don’t know if in reality it will have an effect, you see they want to screw you like a guinea pig’ that's all he told me.—*22 years old*

In practice, PrEP as an HIV prevention method was not used by the majority of respondents and their TW network members, in large part due to these network attitudes toward PrEP. Respondents and other TW in their networks also faced several structural barriers to accessing and using PrEP. The association between PrEP availability and research study participation posed a barrier to PrEP use, not only by contributing to hesitant PrEP attitudes, but also by complicating actual access. For example, several respondents found it tedious and time-consuming to enroll in studies to access PrEP.

Interviewer When you went to [a research center] for PrEP, how did it seem to you?

Respondent Well, I didn’t like it because it was very boring to be there hours and hours, they simply ask you questions and questions… I didn’t want to [enroll in the study] because I realized, they’re going to make me waste my time… because of this I didn’t want to.—*19 years old*

These respondents often made the calculation that the time involved to enroll and participate in research studies could be better spent generating income.

“They took me one time, ‘come on girls to a study to prevent HIV’… and I went and I took the ampule, yeah? …I didn’t finish it and from there I didn’t return ever again… Because we went there… all the girls, by the time they make chats, by the time they do your analysis, you practically spent a whole day and that, for me, kind of annoyed me because they pay little, or rather, I would come tired and didn’t work anymore.”—*26 years old*

Other TW faced additional structural barriers to accessing PrEP because they lacked a legal document of identity (DNI), which further complicates access to general health services, HIV prevention care, and PrEP studies for TW.

Interviewer You haven’t participated in PrEP [study]?

Respondent No, it's that [health promoter] told me that we have to have the document to enter here.

Interviewer The DNI? You don’t have a DNI?

Respondent No, what happened is that I didn’t do the process because I had a judicial problem, I also have been in prison.—*33 years old*

Few respondents that did use PrEP, reported doing so with the encouragement and support of their network members, especially TW who had successfully accessed PrEP themselves. Though this represented a small minority of respondents, when respondents were exposed to TW in their network with positive PrEP attitudes and experiences, they reported feeling more comfortable with PrEP and were sometimes convinced to enroll in studies to access PrEP.

Interviewer And finally, why did you decide to enter the study despite having that fear?

Respondent Because [TW friend] explained to me that no, that this was going to support us and we would take care of ourselves much more.

Interviewer Who had the idea to go to [research center], how did you find out about this study?

Respondent Through [TW friend]—*22 years old*

Health promoters and their messaging were also able to reach and support some respondents to enroll in PrEP studies.

Interviewer Who gave you the information about the pill [PrEP]?

Respondent [health promoter].

Interviewer And then you went to [research center] to do the tests… and they gave you PrEP?

Respondent Yes, the prevention package.—*26 years old*

One respondent was supported and encouraged by her mother to take PrEP. After having her own positive experience with PrEP, she played a role in reassuring others within her network, including her partner.

Respondent Yes, I told him [partner] I was taking PrEP… He told me ‘no, no, don’t take that’… in the beginning he told me ‘don’t take it,’

Interviewer And why did he tell you in the beginning that you shouldn’t take [PrEP]?

Respondent Because he told me, no… you’re like a guinea pig.

Interviewer And he changed his opinion or not that much?

Respondent Once I started to take it I told him that I felt better and he told me, ok that's good… I told him it's to prevent HIV.—*35 years old*

Overall, PrEP uptake and use was uncommon in this cohort. Attitudes and practices related to PrEP tended to be influenced by network members, whether through providing support or voicing hesitancy. Social support for taking PrEP, especially from other TW, contributed to PrEP use among some respondents. However, it was far more common for misinformation and hesitant attitudes surrounding PrEP to be circulated within respondents’ networks. Several structural barriers to PrEP uptake in Peru persist, including the limited access that is primarily controlled by research studies.

## Discussion

We found that TW social networks have the power to influence HIV prevention knowledge, attitudes, and practices, with different relationships representing distinct patterns of influence and information exchange that may be leveraged through a DOI approach to promote PrEP uptake and use. An assessment of the role of TW communities and social networks in reinforcing HIV prevention knowledge, attitudes, and practices, is necessary to design HIV prevention and PrEP outreach efforts that will meaningfully engage TW and support the diffusion of PrEP through TW communities. Our findings improve understanding of the impact of network influence on HIV prevention attitudes and behaviors among TW and highlight important considerations for HIV prevention strategies among TW.

### The Persistent KAP-Gap in HIV Prevention Among TW and the Role of TW Social Networks

Our findings suggest a persistent misalignment between HIV prevention knowledge, attitudes, and practices among respondents (KAP-gap), where different HIV prevention strategies were engaged with differently by TW networks and were met with unique challenges. In general, we found that respondents’ own beliefs and practices were often aligned with other members of their network, including TW friends, family, and primary partners. Routine HIV/STI screening was the only strategy where knowledge and positive attitudes were associated with actual practice. Respondents often shared knowledge and encouragement about accessing HIV/STI screening within their networks and were able to access it themselves or with network support.

Regarding condom use, our findings highlight a potential discrepancy where a high degree of condom knowledge and positive attitudes did not appear to correspond to consistent condom use. Significant contributors to this condom use KAP-gap included barriers to condom use that were outside of respondents’ control such as power imbalances in TW sexual partnerships, lack of HIV/STI prevention knowledge among primary partners, and financial marginalization of TW. While network members such as other TW and family members were considered important for supporting condom use knowledge, information exchange, and positive attitudes, actual condom use was dictated primarily by TW's cis-male sexual partners. Thus, despite the normalization of condom use within networks of TW and their families, social influences and dynamics perpetuated by partners contributed to this KAP-gap in condom use among respondents. Closing this KAP-gap may require that interventions to improve condom acceptability, availability, and use are targeted to sexual partners of TW. In addition, given the power imbalance inherent in condom negotiations within TW partnerships, it is important to consider that condom use alone may not adequately address HIV prevention within this population.^27,39^

PrEP is a strategy with the potential to shift the power dynamics of HIV prevention in favor of TW autonomy. However, TW networks in this study were not defined by the same widespread acceptance of PrEP as of condom use. Although most respondents (85%) were aware of PrEP, knowledge and attitudes surrounding PrEP were primarily characterized by misinformation and mistrust. As a result, there was a notable lack of TW network support for accessing PrEP and very few respondents used PrEP. Several studies have described barriers to PrEP uptake and adherence among TW including PrEP mistrust, prioritization of feminizing hormone therapy and concern over possible interactions, and barriers to accessing healthcare in general—reinforced by social stigma and lack of trans-sensitive care.^8,13,14,40–42^ Our findings suggest that structural barriers to PrEP access—such as the limitation of widespread PrEP availability to research participation—contributed to persistent PrEP hesitancy and lack of PrEP uptake, perpetuating the PrEP KAP-gap.

### Diffusion of Innovations Framework to Address the PrEP KAP-Gap

DOI theory can be used to improve understanding of the current challenges to PrEP uptake and may provide a framework for the introduction and dissemination of PrEP among TW in Peru. Several studies have already documented the presence of behavioral homophily and influential community leaders in TW networks, which is consistent with our study findings, and these DOI concepts could be leveraged to support PrEP dissemination.^24,26,43^ In addition, per DOI theory, the 5 perceived attributes of an intervention that impact its rate of diffusion include its perceived relative advantage, trialability, observability, compatibility, and complexity. With regard to PrEP, some of these attributes can be considered relatively fixed, such as its low trialability, as it cannot be tested on a limited basis before adoption, and low observability, since the primary effect of PrEP (prevention of HIV infection) may not be readily apparent or attributable to PrEP. However, other perceived attributes may be improved with targeted interventions, making DOI theory a potentially useful framework for designing PrEP interventions.

Our findings imply that PrEP is largely incompatible with the existing values and norms of this community of TW, as suggested by the hesitancy and mistrust that emerged in many PrEP discussions among respondents and their networks. Several respondents in our study described a persistent fear of PrEP side effects or lack of awareness of how PrEP functioned, which may further highlight a lack of PrEP outreach that effectively reaches and engages with TW networks, consistent with the current state of PrEP messaging primarily targeted to MSM. In addition, PrEP discussions were defined by significant misinformation while the availability of PrEP through research studies perpetuated notions among TW that PrEP isn’t scientifically proven yet or that they are being used as “guinea pigs.” These findings are consistent with recent literature among TW in Peru and these factors likely contribute to low perceived compatibility of PrEP within this community.^40^

Changing social systems and norms is necessary to improve the perceived compatibility of an intervention and its ultimate diffusion throughout a community. This paradigm shift is typically considered to require a few influential individuals or opinion leaders (“key opinion leaders”) from within the community to begin adopting the intervention, especially for innovations that were initially introduced by individuals outside of the network (ie, foreign researchers).^44–46^ This notion is supported by the findings that some respondents were influenced to try PrEP by other TW who were already using it. PrEP outreach efforts should actively and meaningfully involve TW, and especially TW community leaders, in all steps of the process to create more compatible PrEP messaging and targeted programs that facilitate access for TW. In addition, acceptability research that recognizes the social issues and frameworks that are unique to TW, and does not group TW together with MSM, is necessary to better understand this issue of compatibility.

The inherent barriers to PrEP access posed by the lack of widespread PrEP availability outside of research studies may also contribute to high perceived complexity and low relative advantage of accessing PrEP. Several respondents described the time-consuming process of enrolling and participating in research studies to access PrEP, which, when balanced against the opportunity to spend this time generating income, created a relative financial disadvantage for accessing PrEP. These structural barriers and lack of availability also appeared to contribute to misconceptions and hesitant attitudes surrounding PrEP that were then circulated and reinforced among TW networks. First and foremost, this highlights the need for improved PrEP access outside of clinical research. Adoption of a nationwide PrEP program, such as that in Brazil,^47^ is the first step to removing barriers, decreasing the perceived complexity of using PrEP, and facilitating access.^16^ In addition, the perceived relative advantage of an intervention can sometimes be improved through community campaigns designed with community input. Since PrEP messaging tends to be MSM-centered, interventions that specifically target TW and their networks are needed. Incentives have also been shown to increase perceived relative advantage and facilitate the adoption of an intervention and their potential utility in promoting PrEP uptake among TW should be considered.^48,49^

Leveraging TW network input, empowering TW at the community-level, designing specific programs for this context, and calling for widespread access to PrEP through national public health systems may improve the perceived compatibility, relative advantage, and complexity associated with using PrEP in order to improve diffusion of PrEP through TW communities. However, it is also important to understand the limitations of DOI theory in this setting. First, this theory has been criticized for not taking into account an individual's resources or social support to adopt a behavior.^50^ Our findings show that social support may play a substantial role in promoting HIV prevention and ultimately facilitating access to HIV prevention tools among TW. Therefore, we cannot ignore the importance of social networks in potentially improving the uptake of PrEP. DOI theory has also been criticized for focusing heavily on the agency of the individual to adopt a behavior while ignoring institutional constraints and the broader, systemic context.^50,51^ Again, our findings and prior studies show that existing systems create structural barriers to accessing HIV care and PrEP, even when individual motivation to access these resources may be high.^13,52^ These barriers should be acknowledged and addressed in order to achieve adequate diffusion of PrEP interventions. Despite the limitations of DOI theory, DOI concepts still have value as guiding principles to design more effective PrEP interventions and promote the uptake of PrEP in this context.

This study has additional limitations that should be considered. First, given the specific social and cultural context of this sample of TW in Lima, Peru, our findings might not be generalizable to TW in other regions of Peru or Latin America. In addition, though PrEP access for most TW in Peru continues to be practically limited to research participation, PrEP awareness, perceptions, and patterns of information dissemination among TW communities may have changed since data were collected in 2018. Though our study sheds light on the role TW play in HIV prevention knowledge sharing across their networks, additional research is needed to elaborate on the ways in which PrEP information is currently introduced to and shared within TW communities and how PrEP attitudes may have changed. Despite these limitations, our findings contribute to an improved understanding of the role of TW social networks in influencing HIV prevention knowledge, attitudes, and practices and have important implications for future PrEP programs aiming to improve PrEP uptake in TW communities.

## Conclusions

TW play an important role in disseminating and reinforcing HIV prevention knowledge, attitudes, and norms within their networks. TW's diverse networks are instrumental sources of support and have the potential to influence HIV prevention beliefs and practices. PrEP outreach interventions and messaging should specifically target TW and leverage community relationships to improve PrEP acceptability and access. HIV prevention research and interventions in Latin America have historically considered TW a subpopulation of MSM in research study and program design.^13^ Though there has been recent progress in this area, currently, most Latin America countries, including Peru, do not have tailored HIV prevention programs for TW. Interventions can consider utilizing DOI theory as a guiding framework to improve PrEP diffusion through TW communities. Interventions and research should also be designed with TW input in order to prioritize compatibility with existing norms and needs of the community and to promote peer support, empowerment, and knowledge-sharing. Such interventions can also consider ways to include family and partners in outreach efforts. Finally, changes to public health policy are necessary to remove structural barriers to PrEP access and promote institutional reform to improve TW's experiences with healthcare services.
